# Comparison of genetic variation between northern and southern populations of *Lilium cernuum* (Liliaceae): Implications for Pleistocene refugia

**DOI:** 10.1371/journal.pone.0190520

**Published:** 2018-01-04

**Authors:** Mi Yoon Chung, Son Hai Vu, Jordi López-Pujol, Sonia Herrando-Moraira, Sungwon Son, Gang Uk Suh, Hoa Thi Quynh Le, Myong Gi Chung

**Affiliations:** 1 Department of Biology and the Research Institute of Natural Science, Gyeongsang National University, Jinju, Republic of Korea; 2 BioC-GReB, Instituto Botánico de Barcelona, IBB, CSIC-ICUB, Barcelona, Spain; 3 Plant Conservation Division, Korea National Arboretum, Pocheon, Republic of Korea; National Cheng Kung University, TAIWAN

## Abstract

The so-called “Baekdudaegan” (BDDG), a mountain range that stretches along the Korean Peninsula, has been recently proposed as a major “southern” glacial refugium for boreal or temperate plant species based on palaeoecological and, especially, genetic data. Genetic studies comparing genetic variation between population occurring on the BDDG and more northern ones (i.e. in NE China and/or in Russian Far East) are, however, still too few to draw firm conclusions on the role of the BDDG as a refugium and a source for possible northward post-glacial recolonizations. In order to fill this gap, we selected a boreal/temperate herb, *Lilium cernuum*, and compared levels of allozyme-based genetic diversity of five populations from NE China with five populations from South Korea (home of its hypothesized refuge areas). As a complementary tool, we used the maximum entropy algorithm implemented in MaxEnt to infer the species’ potential distribution for the present time, which was projected to different past climate scenarios for the Last Glacial Maximum (LGM). Permutation tests revealed that Korean populations harbored significantly higher levels of within-population genetic variation than those from NE China (expected heterozygosity = 0.173 vs. 0.095, respectively). Our results suggest that the lowered levels of genetic diversity in NE Chinese populations might be due to founder effects associated with post-glacial migration from southern regions. Congruent with genetic data, past distribution models showed higher probability of occurrence in southern ranges than in northern ones during the LGM. In addition, a positive correlation was detected between the expected heterozygosity and environmental LGM suitability. From a conservation perspective, our results further suggest that the southern populations in South Korea may be particularly worthy of protection.

## Introduction

Unglaciated regions and/or regions that provided relatively stable environmental conditions during the Last Glacial Maximum (LGM; ca. 21,000 yr BP) are generally regarded as candidates for glacial refugia for plant species, at least in the Northern Hemisphere [1–4]. Therefore, it can be anticipated that populations located within refugia would show high levels of genetic diversity, as plant species would have endured the Pleistocene harsh climatic conditions by maintaining relative large populations there. Furthermore, plant populations in refugial areas would have had a longer demographic history than those that are result of post-glacial colonization because the latter would have experienced recurrent bottlenecks/founder effects [5]. In the formerly glaciated areas, on the contrary, low levels of genetic diversity are expected, as populations are of recent origin (originated through recolonization following deglaciation and, thus, suffering from founder effects).

In agreement with these expectations, a large body of studies—especially conducted in Europe—have revealed a common pattern of latitudinal decrease of genetic variation, which is often referred to as “southern richness vs. northern purity” [2,6,7,8,9]. Such studies are also abundant in North America (e.g. [10,11]), especially in the eastern coast of the United States where there is one of the most studied refugia of the world, the Southern Appalachians (see [12] for a review). There are many examples of higher levels of genetic diversity in plant populations of the Southern Appalachians compared to more northern conspecific ones, with several cases showing a south-to-north decrease of genetic diversity (e.g. [13,14]). In addition, several congeneric comparisons have consistently revealed lower genetic variability in northern species compared to their southern congeners [15–18].

On the Korean Peninsula, the so-called “Baekdudaegan” (hereafter the “BDDG”) is undeniably one of the most important mountain systems in East Asia in terms of length, ecological value, and sacredness [5,12]. More specifically, the BDDG is the longest mountain chain of NE Asia, stretching ca. 1625 km in a north-to-south direction, and it is the major center of plant diversity of the Korean Peninsula, harboring over 1500 taxa. In addition, it contains sites holy to all the major religions present in the country, namely Shamanism, Buddhism, Daoism, Neo-Confucianism, and even Christianism [5,12]. More significantly, it has been recently proposed as a major Pleistocene refugium for the boreal and temperate flora of NE Asia based on both palaeoecological and genetic evidence [5]. First, all the high-resolution pollen records available for the peninsula unambiguously suggest that most of the BDDG was covered by boreal or mixed boreal/temperate forests, a scenario also supported by several palaeovegetation reconstructions ([19–22]; but see [23,24]). Second, a series of allozyme genetic diversity studies conducted in boreal and/or temperate plant species mostly centered in the southern part of the BDDG has revealed a pattern of high within-population and low to moderate among-population genetic variability (Table 1 in [5]), suggesting that the BDDG acted as a refugium for these plant species. However, there are still very few comparisons between populations of boreal and/or temperate plant species occurring on the BDDG and populations farther north (i.e. in NE China and/or in Russian Far East).

Broadly, two scenarios could be proposed in relation to the levels of within-population genetic variation of boreal/temperate plant species that are native to the BDDG but that also occur in more northern latitudes. If a given species vanished from northern areas during the LGM and then recolonized the area through post-glacial migration from its putative refugia in the BDDG (see below), we may expect diminished levels of genetic diversity in populations located at more northerly latitudes, due to founder effects. Alternatively, if the species would have also endured the LGM *in situ* in northern areas, we may expect similar levels of genetic diversity in northern and southern populations.

**Fig 1:**
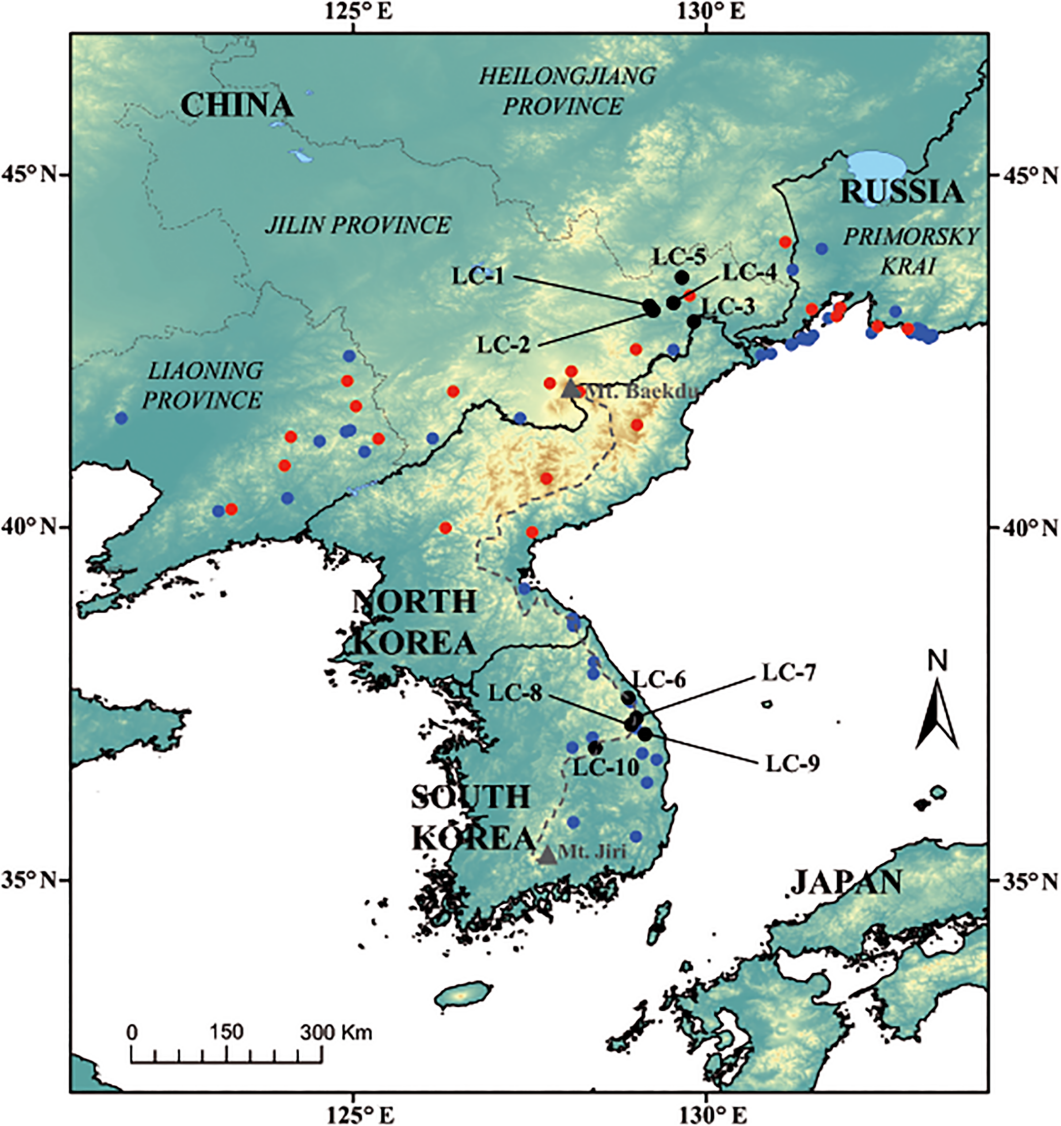
Location of sampled populations of *Lilium cernuum* (black circles, LC-1 to LC-5 from NE China; LC-6 to LC-10 from South Korea). Grey dashed line indicates the approximate location of the ridge of the main mountain range of the country, the so-called “Baekdudaegan” (BDDG), which runs north to south along the Korean Peninsula. The blue circles are precise occurrence records (which correspond to those used in ecological niche modeling; see Fig 5), whereas the red circles are approximate localities of *L*. *cernuum*. Presence records, regardless of their precision, have been mostly obtained from herbarium specimens but also from databases, scientific papers, and grey literature.

In this study, we tested these alternative hypotheses with the boreal/temperate and shade-tolerant herb *Lilium cernuum* Kom. (Liliaceae). We believe that this is a good study system because of the following reasons. First, the species is distributed from Primorsky Krai in Russian Far East, Jilin and Liaoning provinces in NE China to the south of the Korean Peninsula ([25]; Fig 1); that is, the species’ range covers a latitudinal stretch of nearly 10°. And, second, a previous study conducted in the southern section of the BDDG and in its main branch (the so-called “Nakdongjeongmaek”) reported high within-population genetic variation [26,27], suggesting that this region was a putative glacial refugium for the species. We have selected a set of populations from the BDDG and a set of populations located much further north, in Jilin (NE China) and compared their genetic variability. As a complementary tool to the genetic study, we have used ecological niche modeling (ENM) to get insights into the palaeodistribution (at the LGM) of *L*. *cernuum* and to see whether there is congruence between ENM and genetic data.

## Materials and methods

### Study plant

*Lilium cernuum* is a herb 30–80 cm tall, with scattered, narrowly linear leaves. Pale purple-red flowers (ca. 4.0 cm long) are solitary or arranged in racemes of 2–7, nodding, and fragrant, and open in July. Fruits (capsules) are globose to obovoid, 1.5 cm long. The breeding system and pollinators are unknown [27]. It is a diploid species (2*n* = 24; http://ccdb.tau.ac.il/Angiosperms/Liliaceae/Lilium/Lilium%20cernuum%20Ko) that occurs in thickets and sunny grassy slopes in the northern part of its range (NE China, Russian Far East; [25,28]) but under *Quercus mongolica*-dominated temperate deciduous forests in Korea [29]. It is regarded as threatened both in Russia [30] and China [31] but not in Korea.

*Lilium cernuum* is considered endangered in China [it is listed as “VU” (“Vulnerable”) in the Red List of 2013] but, to our knowledge, it is not protected because it was not included in the laws of 1984 (the so-called “National List of Rare and Endangered Plant Species”) and 1999 (the “Catalogue of the National Protected Key Wild Plants”). In South Korea, *L*. *cernuum* was delisted as an endangered species in 2012. Now it has been listed as “LC” (“Least Concern”), i.e., not considered threatened.

We selected five populations from NE China (*n* = 207) and five populations from South Korea (*n* = 217), and these were sampled from areas of similar size to prevent area effects (Fig 1). None of these populations were inside nature reserves. In each population, we collected samples randomly from adults (flowering individuals). To minimize the damage to these lilies, we collected only one leaf per individual. Korean populations (LC-6 to LC-10) correspond to the LC-2 to LC-6 populations of the former study of 2014 [27], although the sample sizes of the present study are larger (mean *n* = 43 vs. 34). In addition, the resolved allozymes are not the same (*Mdh-1* and *Mdh-2* are lacking in the present study as the resolution of banding patterns was not optimal for the NE China populations).

### Enzyme electrophoresis

For enzyme extraction, we crushed leaf samples using chilled mortars and pestles and an extraction buffer [32] to solubilize and stabilize the enzymes. We absorbed enzyme extracts onto 4 × 6 mm paper wicks (Whatman 3MM chromatography paper) and we stored them at −70°C until needed. We conducted electrophoresis on 13% starch gels, with two buffer systems (Table 1). We stained gels for eight enzyme systems, which were alcohol dehydrogenase (ADH, E.C.1.1.1.1), diaphorase (DIA, E.C.1.6.99.1), fluorescent esterase (FE, E.C.3.1.1.1), isocitrate dehydrogenase (IDH, E.C.1.1.1.42), phosphoglucoisomerase (PGI, E.C.5.3.1.9), 6-phosphogluconate dehydrogenase (6PGD, E.C.1.1.1.44), phosphoglucomutase (PGM, E.C.2.7.5.1), and triosephosphate isomerase (TPI, E.C.5.3.1.1). We utilized stain recipes from [33], except for DIA [34]. We designated putative loci designated sequentially, with the most anodally migrating isozyme designated as *1* and the next *2*. We also designated different alleles per locus sequentially by alphabetical order.

**Table 1:** The allozyme loci assayed for *Lilium cernuum* and the buffer systems used to resolve them^a^. Buffer system no. 34 was a modification [35] of the system 6 of [33]. The morpholine-citrate system (MC, pH 6.1) was from [36].

Buffers system	Loci resolved
34	*Adh*, *Dia-1*, *Dia-2*, *Fe*, *Pgi-1*, *Pgi-2*, *Pgm*, *Tpi-1*, *Tpi-2*
MC	*Idh*, *6Pgd-1*, *6Pgd-2*

^a^ Abbreviations for the loci are given in the materials and methods section.

### Data analyses

To estimate genetic diversity and structure, we considered that a locus was polymorphic when two or more alleles were observed, regardless of their frequencies. We estimated the following genetic diversity parameters using the programs POPGENE [37] and FSTAT [38]: percent polymorphic loci (*%P*), mean number of alleles per locus (*A*), allelic richness (*AR*) using a rarefaction method that compensates uneven population sample sizes [39,40], observed heterozygosity (*H*_o_), and Nei [41]’s unbiased gene diversity or Hardy–Weinberg (H–W) expected heterozygosity (*H*_e_). Hereafter, the subscript “s” indicates species’ (or pooled samples) values, while the subscript “p” indicates population means.

To test for differences between populations in South Korea and those in NE China for observed statistics, *OSx* (*AR*, *H*_o_, and *H*_e_), we used a permutation scheme (999 replicates) by randomly allocating whole samples to the different groups, keeping the number of samples in each group constant and calculating differences between South Korean and northern Chinese populations for randomized statistics, *RSx*. We then obtained the *P* value of the test as the proportion of randomized data sets giving higher values for *RSx* than for *OSx*. These calculations were performed using FSTAT [38].

To test for recent decreases in effective population size (bottlenecks), we evaluated for individual loci the difference between the H–W *H*_e_ and the equilibrium heterozygosity (*H*_eq_) expected assuming mutation–drift equilibrium. These differences were evaluated using a sign test and a Wilcoxon sign-rank test conducted across loci under an infinite allele model using the program BOTTLENECK [42]. Since allelic diversity is generally lost more rapidly than *H*_e_ [43], recently bottlenecked populations are expected to exhibit an excess of H–W equilibrium *H*_e_ relative to *H*_eq_ [44,45].

We estimated population-level *F*_IS_ (inbreeding) and calculated its significance level (*P* values) by gene permutation tests (999 replicates) under the null hypothesis (*F*_IS_ = 0) using the program SPAGeDi [46]. We also calculated Wright [47]’s *F*_IS_ and *F*_ST_ over loci following [48]. These fixation indices measure the average deviation from H–W equilibrium of individuals relative to their local populations (*F*_IS_, a measure of local inbreeding) and local populations relative to the total population (*F*_ST_, a measure of differentiation between local populations). The significance of multi-population *F*_IS_ and *F*_ST_ estimates was determined by a permutation test (999 randomizations of alleles between individuals within samples and 999 randomizations of genotypes between populations, respectively). These calculations were performed using FSTAT [38]. Statistical significance of differences in *F*_IS_ and *F*_ST_ between populations in southern Korea and those in NE China was determined as outlined above for *AR*, *H*_o_, and *H*_e_. To test for the influence of individuals within populations, populations within regions, and regions on the observed genetic variation, we conducted an analysis of molecular variance (AMOVA) using the program GenAlEx 6.5 [49].

A UPGMA (unweighted pair-group method using arithmetic averages) phenogram was generated from Nei et al. [50] genetic distance matrix with branch support produced by 1000 bootstrapping over loci, utilizing Populations 1.2.30 [51] and TreeView 1.6 [52]. We assessed the genetic structure by means of the Bayesian algorithm implemented in STRUCTURE 2.3.4 [53]. The program estimates the likelihood of the individuals being structured in a given number of genetic clusters (or genetic populations, *K*). The admixture ancestry model with correlated allele frequencies was selected as an appropriate option for the analysis, as it is the best combination for dealing with species with presumably complex dynamics; individuals with mixed ancestry, events of migration, and populations with shared ancestry are presumably to occur in *L*. *cernuum*. The burn-in period and Markov Chain Monte Carlo (MCMC) were set to 50,000 and 500,000 iterations, respectively, and 10 replicates per *K* were run, with a priori grouping of individuals into populations. The most likely value of *K* was determined both by choosing the smallest *K* after the log probability of data [ln Pr(*X|K*)] values reached a plateau [53] and by the Δ*K* statistic of [54], with the aid of STRUCTURE HARVESTER [55]. For the most likely *K*, Clumpp 1.1.2b [56] was used to combine the results of the 10 replicates of the best *K*. To plot the output result produced by Clumpp, we used the program Distruct 1.1 [57].

Finally, we investigated whether there was isolation-by-distance by conducting a Mantel test between all pairwise *F*_ST_/(1 –*F*_ST_) values (*F*_ST_ was calculated following [48]) and the corresponding logarithm of pairwise geographical distances [58]. A linear regression model under the null hypothesis of no spatial genetic structure (regression slope, *b* = 0) was tested (by making 999 replicates) using the program Permute![59].

### Ecological niche modeling (ENM)

Ecological niche modeling (ENM) was performed to evaluate the potential distribution of *L*. *cernuum* under present climatic conditions and to project it to the LGM. We employed the maximum entropy algorithm, as implemented in MaxEnt 3.3 [60]. The current distribution information for *L*. *cernuum* was obtained from: (1) major databases and information systems, including the Global Biodiversity Information Facility (GBIF; http://www.gbif.org/) and the Chinese Virtual Herbarium (http://www.cvh.ac.cn); (2) research articles, books, and grey literature (i.e. theses (e.g. [61]) and unpublished reports); (3) citizen science projects, such as iNaturalist (http://www.inaturalist.org), Chinese Field Herbarium (CFH; http://www.cfh.ac.cn), and Plant Photo Bank of China (PPBC; http://www.plantphoto.cn); and (4) the sampling sites of this study (Table 2). In total, after removing duplicate records within each pixel (2.5 arc-min, ca. 5 km), we obtained 58 presences. A set of 19 bioclimatic variables at 2.5 arc-min resolution covering the species distribution range (and neighboring areas) under current conditions (1950–2000) were downloaded from the WorldClim website (http://www.worldclim.org; [62]). After a pairwise correlation analysis, carried with the SDM Toolbox extension for ArcMap [63], we selected a smaller set of six (relatively) uncorrelated variables: mean diurnal range (bio2), isothermality (bio3), mean temperature of the wettest quarter (bio8), mean temperature of the coldest quarter (bio11), precipitation of the wettest month (bio13), and precipitation of the driest month (bio14). The selection of variables from pairs or groups of highly correlated (*r* ≥ |0.8|) ones was done on the basis of their relative contribution to the model (percent contribution, jackknife tests of variable importance) and the shape of their response curves.

**Table 2:** Summary of genetic diversity measures and mean fixation values (*F*_IS_) for five Chinese populations and five Korean populations of *Lilium cernuum*^a^.

Pop	Alt (m)	*n*	*%P*	*A*	*AR*	*H*_o_ (SE)	*H*_e_ (SE)	*F*_IS_
Northern populations (NE China)								
LC-1	888	26	41.7	1.67	1.67	0.093 (0.039)	0.126 (0.055)	0.288^b^
LC-2	356	48	41.7	1.58	1.53	0.056 (0.025)	0.085 (0.041)	0.344^b^
LC-3	259	38	50.0	1.75	1.71	0.085 (0.038)	0.108 (0.047)	0.212^b^
LC-4	453	33	41.7	1.67	1.64	0.063 (0.030)	0.075 (0.036)	0.153
LC-5	459	62	41.7	1.50	1.46	0.055 (0.026)	0.084 (0.040)	0.343^b^
Average	483	41	43.3	1.63	1.60	0.070 (0.008)	0.095 (0.009)	0.280^c^
Southern populations (South Korea)								
LC-6	1020	52	58.3	2.00	1.93	0.143 (0.053)	0.161 (0.059)	0.116^b^
LC-7	960	43	58.3	1.92	1.88	0.182 (0.063)	0.201 (0.065)	0.094
LC-8	1028	34	75.0	1.92	1.90	0.179 (0.049)	0.208 (0.053)	0.140^b^
LC-9	1010	51	41.7	1.67	1.58	0.137 (0.006)	0.170 (0.070)	0.191^b^
LC-10	1220	37	50.0	1.67	1.66	0.106 (0.046)	0.124 (0.049)	0.145^b^
Average		43	56.7	1.84	1.79	0.149 (0.014)	0.173 (0.015)	0.136^c^
Species level		424	75.0	2.33		0.109 (0.036)	0.152 (0.053)	0.185^c^

^a^ Abbreviations: *Pop* population, *Alt* (m) altitude (m) a.s.l., *n* the number of individuals sampled, *%P* percentage of polymorphic loci, *AR* mean allelic richness (adjusted for a sample size of 26 in LC-1), *A* mean number of alleles per locus, *H*_o_ observed heterozygosity, *H*_e_ H–W expected heterozygosity or genetic diversity, *SE* standard error, *F*_IS_ fixation index within populations.

^b^ Significance (*P* < 0.05) based on permutation (999 replicates) under the null hypothesis of *F*_IS_ = 0.

^c^ Significant (at the 0.05 level) Weir and Cockerham [48] estimate of *F*_IS_ over populations.

The distribution model under current conditions was projected to the LGM using palaeoclimatic layers simulated by both the Community Climate System Model Version 4 (CCSM4; [64]), the Model for Interdisciplinary Research on Climate Earth System Model (MIROC-ESM; [65]), and the New Earth System Model of the Max Planck Institute for Meteorology (MPI-ESM-P: http://www.mpimet.mpg.de/en/science/models/mpi-esm/). Replicate runs (20) of MaxEnt using the “subsample” method were performed to ensure reliable results. Model performance was assessed using the area under the curve (AUC) of the receiver operating characteristic plot, with 25% of the localities randomly selected to test the model. AUC scores may range between 0.5 (randomness) and 1 (exact match), with those above 0.9 indicating a good performance of the model [66]. The MaxEnt jackknife analysis was used to evaluate the relative importance of the six bioclimatic variables employed, based on their gain values when used in isolation. In a simple way to reduce the uncertainty associated to the use of different global climate models (GCMs), we produced a LGM-ensemble map that identified all areas predicted as suitable that were common to the three LGM projections. To do this, firstly we converted the three continuous projection maps into binary (presence/absence) distribution maps, applying the maximum sensitivity plus specificity logistic threshold, which is very robust with all types of data [67]. Secondly, we overlapped the binary output maps with the Intersect Tool of ArcGIS. All ENM predictions were visualized in ArcGIS 10.2 (ESRI, Redlands, CA, USA).

### Comparison between genetic diversity and habitat suitability

In biogeography, ENM is used as a complementary tool to the genetic markers in order to reconstruct species palaeodistributions, often showing good agreement (e.g. [68,69]). Herein, following a methodology that is based on [70], we analyzed the relationship between *H*_e_ and habitat suitability (measured as MaxEnt’s logistic output, a value that ranges from 0 to 1 and that can be cautiously interpreted as the predicted probability of presence; see [71]) by means of Pearson’s correlations, for all ten studied populations of *L*. *cernuum*. Correlations were performed separately for the present time and for the LGM; for this latter, the values of habitat suitability were obtained by averaging the values for each of the three palaeoclimatic scenarios (CCSM4, MIROC-ESM, and MPI-ESM-P).

## Results

### Genetic diversity in northern and southern populations of *Lilium cernuum*

Of the 12 putative loci surveyed for *L*. *cernuum* (Table 2), nine (*Adh*, *Dia-1*, *Fe*, *Idh*, *6Pgd-1*, *6Pgd-2*, *Pgi-2*, *Pgm*, and *Tpi-1*) were polymorphic across 10 populations, resulting in high levels of genetic variation in pooled samples (*n* = 410, *%P*_s_ = 75.0, *A*_s_ = 2.33, and *H*_es_ = 0.152; Table 2). Considerably higher levels of genetic variation were found within southern populations (South Korea) compared to northern populations (NE China): *%P*_p_ = 56.7 vs. 43.3, *A*_p_ = 1.84 vs. 1.63, *AR* = 1.79 vs. 1.60, and *H*_ep_ = 0.173 vs. 0.095 (Table 2). Accordingly, we found significant differences for *AR*, *H*_op_, and *H*_ep_ between South Korean and NE Chinese populations (one-sided *P*-values were 0.043, 0.002, and 0.006, respectively). Regarding the allelic composition, we identified 22 alleles in the northern populations and 28 alleles in the southern ones; six alleles were exclusive to the Korean populations and none of them to the Chinese ones (i.e. the Chinese populations could be considered a subset of the allelic diversity of the Korean ones; S1 Table). Among the 10 studied populations, there were no statistically significant signals of recent bottlenecks (S2 Table).

### Inbreeding and population genetic structure

We found a significant deficiency of heterozygotes (at the 0.05 level) relative to H–W expectations in all but LC-4 and LC-7 populations (Table 2). These results, as well as the significant multi-population-level *F*_IS_ (*F*_IS_ = 0.185, *P* = 0.001; Table 2), indicated a substantial deficit of heterozygotes within populations. The values of pooled multi-population *F*_IS_ for northern and southern populations were significantly greater than zero (*F*_IS_ = 0.280, *P* = 0.001 vs. *F*_IS_ = 0.136, *P* = 0.001; Table 2), but the former was significantly larger than the latter (*P* = 0.001). Deviation from H–W expectations due to allele frequency differences between populations were, despite low, significantly different from zero for the two regions (*F*_ST_ = 0.108, *P* = 0.001 for northern populations vs. *F*_ST_ = 0.094, *P* = 0.001 for southern populations). However, we found no statistically significant differences between the two groups of populations (*P* = 0.571). Across 10 populations, the value of *F*_ST_ was 0.133 (*P* = 0.001). Consistent with these results, the AMOVA indicated that most of the variation resided within populations (84%), with variation attributable to differences between regions (South Korea vs. NE China) and among populations within regions being only 7% and 9%, respectively (Table 3).

**Table 3:** Analysis of molecular variance (AMOVA) of *Lilium cernuum* populations^a^.

Source of variation	df	SS	MS	VC	Variation (%)
Between regions (NE China vs. South Korea)	1	35.154	35.154	0.062	7
Among populations within regions	8	65.989	8.249	0.089	9
Within populations	838	667.838	0.797	0.797	84
Total	847	768.981		0.948	100

^a^ Abbreviations: *df* degrees of freedom, *SS* sum of squares, *MS* mean squares, *VC* variance components.

**Fig 2:**
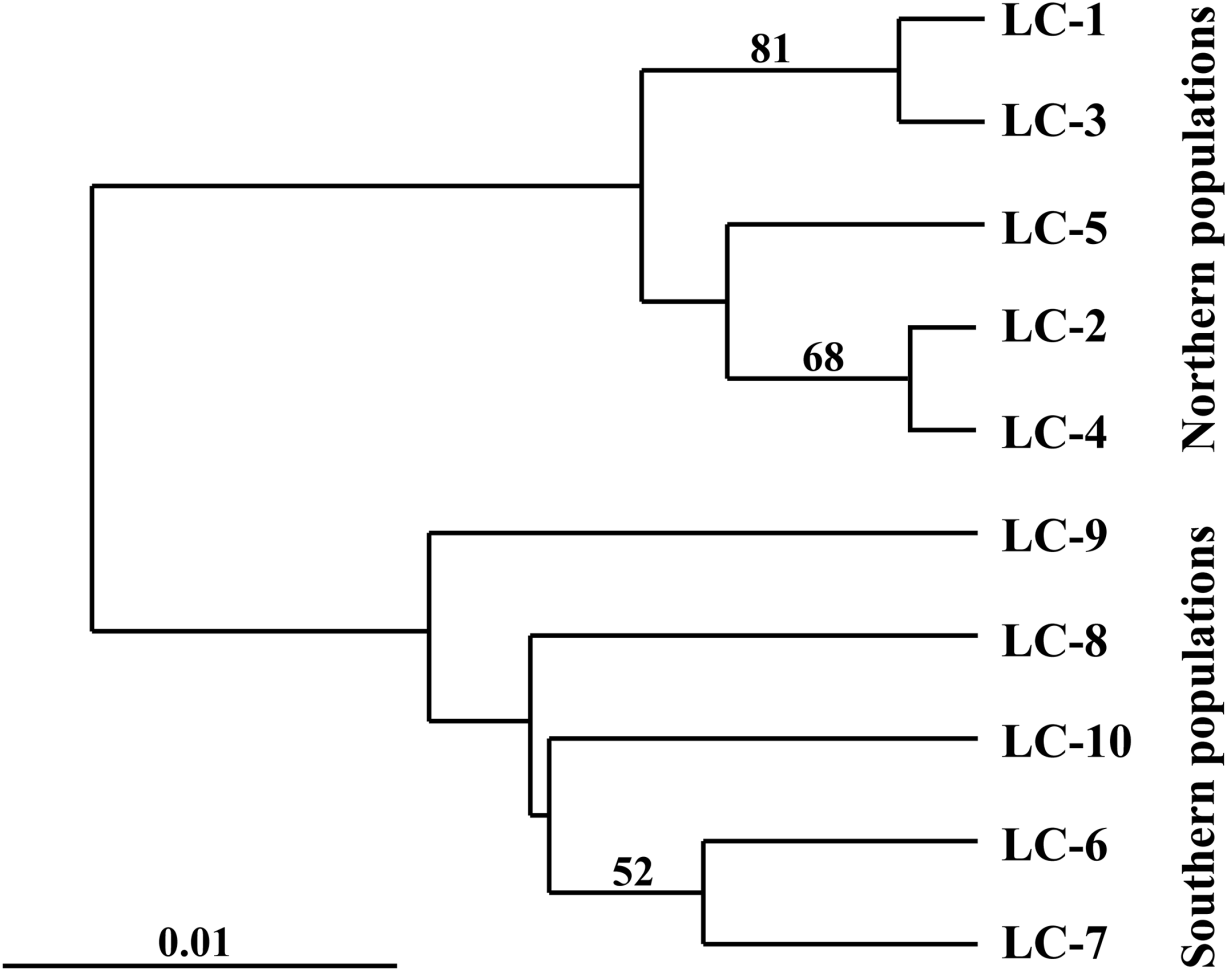
UPGMA phenogram based on Nei et al. [50] genetic distances between populations of *Lilium cernuum*. Note that LC-1 to LC-5 are from NE China, whereas LC-6 to LC-10 are from South Korea. Bootstrap values greater than 50% from 1000 replicates were found only for three branches.

**Fig 3:**
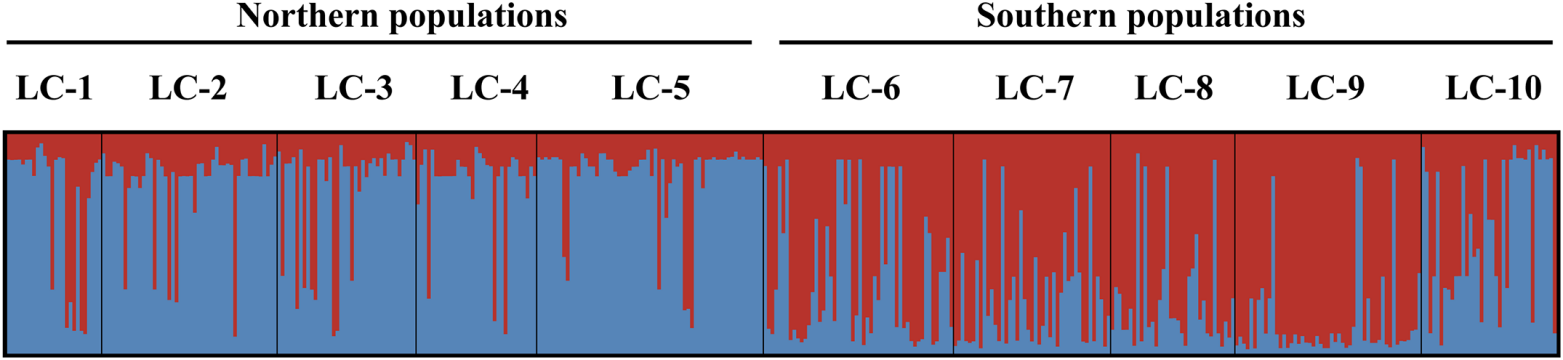
Results of STRUCTURE for all *Lilium cernuum* studied individuals. Assignation of individuals to genetic clusters is at *K* = 2.

The UPGMA phenogram (Fig 2) revealed a clear genetic separation between northern and southern populations. The best clustering scheme of STRUCTURE (*K* = 2, according to both the ln Pr(*X*|*K*) and the Δ*K* statistic; S1 Fig), agreed with the UPGMA phenogram, although some populations, such as LC-1, and especially LC-10, showed a high degree of admixture (Fig 3).

**Fig 4:**
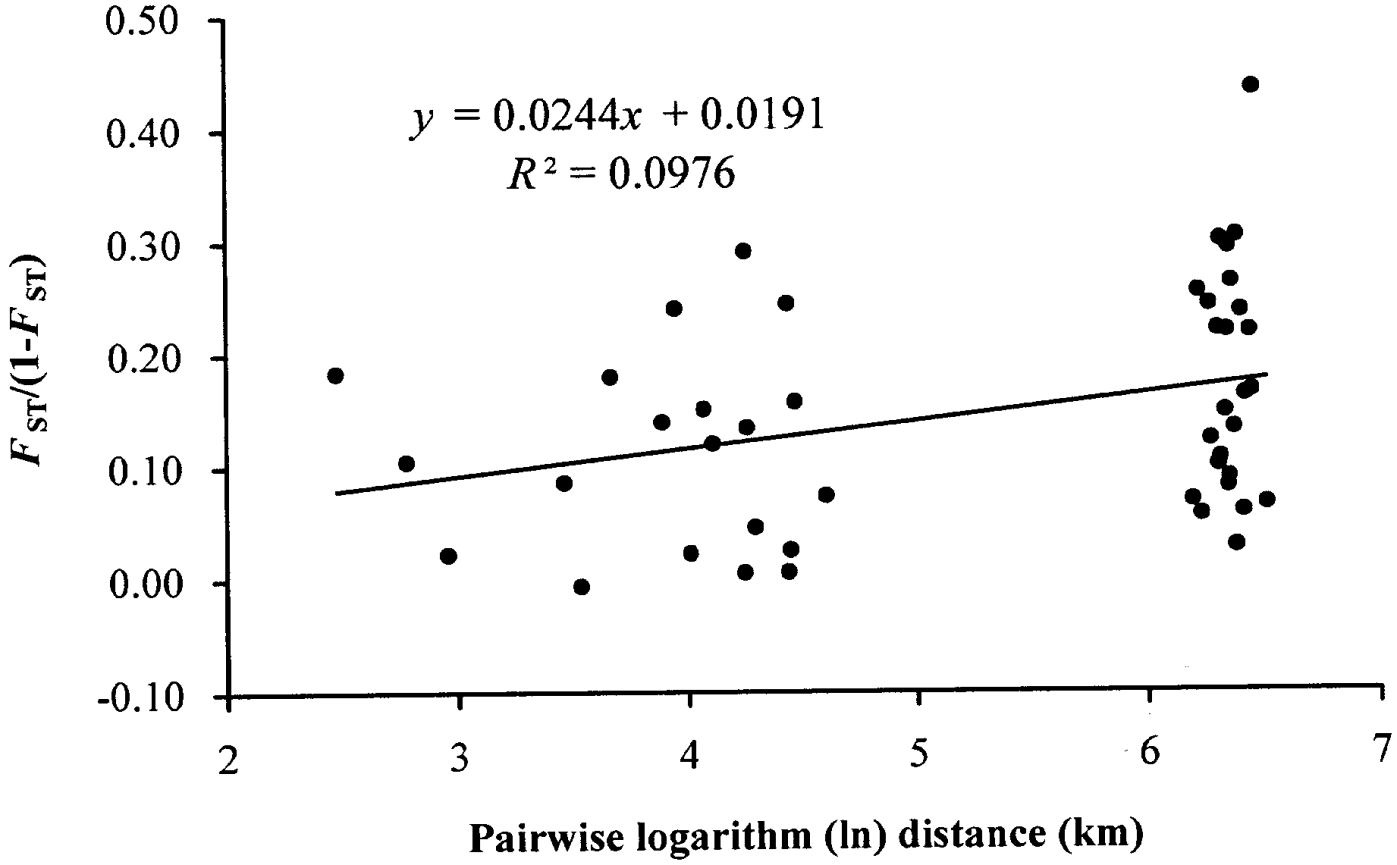
Differentiation between populations of *Lilium cernuum*. Multilocus estimates of pairwise differentiation of *F*_ST_/(1 − *F*_ST_) are plotted against logarithm (ln) of pairwise geographic distances in meters. A significant relationship was found for *L*. *cernuum* (*r* = 0.312, *P* = 0.041).

Finally, we found a significant positive linear relationship between pairwise *F*_ST_/(1 –*F*_ST_; see Table 4 for pairwise *F*_ST_ estimates) and logarithm of pairwise linear geographic distances (*r* = 0.312, *P* = 0.041; Fig 4).

**Table 4:** Pairwise *F*_ST_ values between 10 populations of *Lilium cernuum*. The significant *F*_ST_ values are in **bold**.

Populations	LC-1	LC-2	LC-3	LC-4	LC-5	LC-6	LC-7	LC-8	LC-9
LC-2	**0.155**								
LC-3	0.006	**0.132**							
LC-4	**0.153**	-0.006	**0.123**						
LC-5	**0.025**	**0.197**	0.006	**0.194**					
LC-6	**0.054**	**0.204**	**0.065**	**0.197**	**0.082**				
LC-7	**0.097**	**0.182**	**0.110**	**0.181**	**0.141**	**0.022**			
LC-8	**0.076**	**0.130**	**0.092**	**0.118**	**0.143**	**0.044**	**0.022**		
LC-9	**0.210**	**0.228**	**0.232**	**0.234**	**0.304**	**0.137**	**0.079**	**0.095**	
LC-10	**0.056**	**0.193**	**0.028**	**0.181**	**0.062**	**0.069**	**0.119**	**0.108**	**0.226**

### Ecological niche modeling and congruence with genetic data

**Fig 5:**
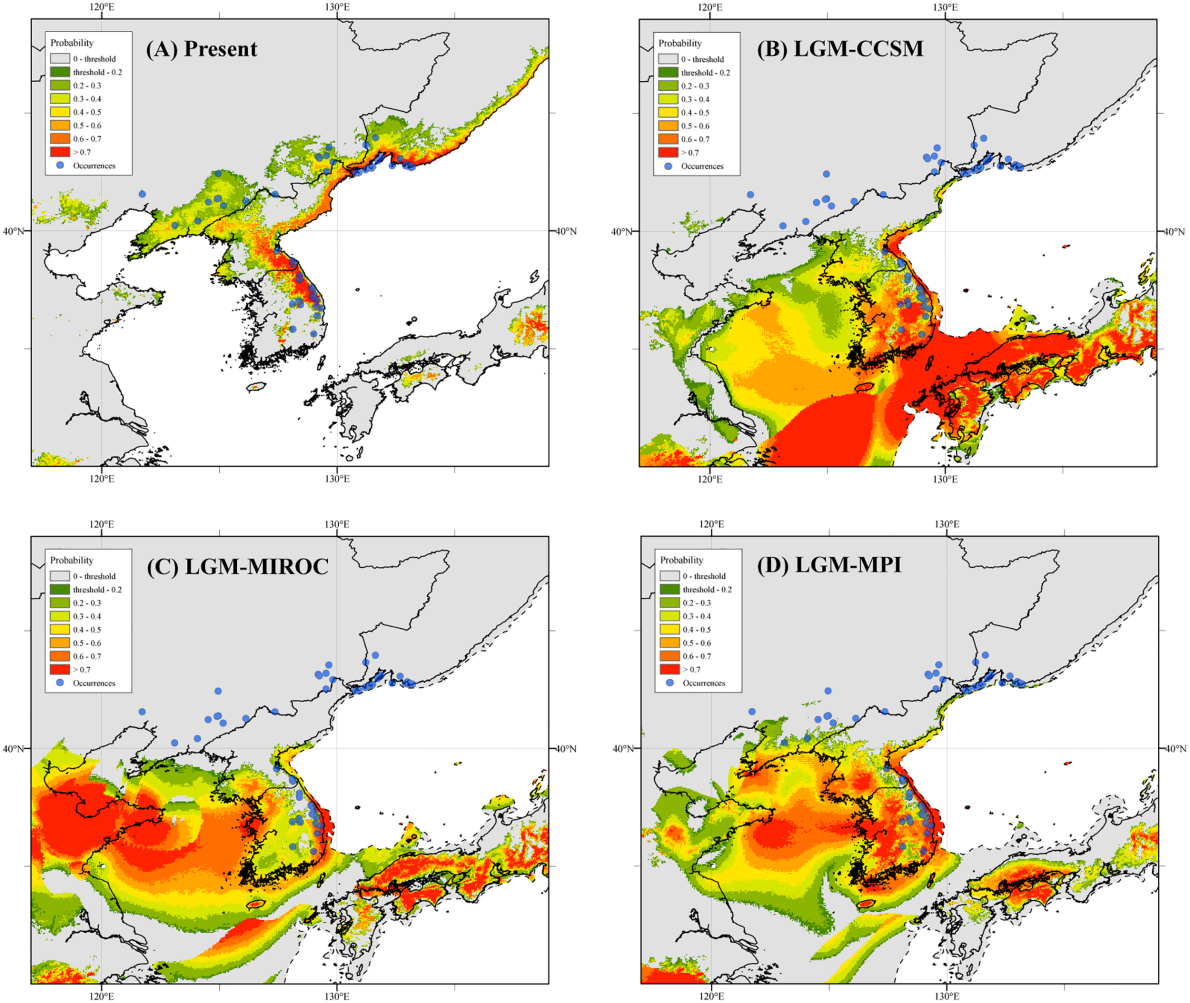
A comparison of potential habitats for *Lilium cernuum* under the present climate and three climatic scenarios for the Last Glacial Maximum (LGM, ca. 21,000 yr BP). (A) Potential habitats under the present climate; (B) Potential habitats expected for the LGM using the CCSM4 model, (C) the MIROC-ESM model, and (D) the MPI-ESM-P model. The darker color indicates a higher probability of occurrence. Maps were generated using the software ArcGIS 10.2. The reconstructed LGM coastlines are represented as dashed lines. Blue circles are the precise occurrence records of *L*. *cernuum* (at 2.5 arc-min resolution) used to build the models.

The AUC scores averaged across 20 runs were high (> 0.93) for the four models built (present only, and present projected to CCSM4, MIROC-ESM, and MPI-ESM-P), which supported their predictive power. According to the MaxEnt jackknife tests of variable importance, the most informative variables for all models were bio11, bio14, and bio8 (in this order). The present day distributional predictions for *L*. *cernuum* were largely congruent with the known occurrences; the large areas of North Korea without occurrences are mostly due to the lack of floristic research since the 1950s and not to the actual absence of the plant there. Projections of the species niche to the LGM climate produced considerably different maps of probability of occurrence compared to the present time. All three models, although variable, showed a similar pattern of southwards displacement of the suitable areas; such areas included the southern portion of North Korea, most of South Korea, a large part the exposed East China Sea shelf (including the Yellow Sea), large parts of the Korea Strait, and southern Japan (Fig 5). Indeed, the LGM-ensemble map hardly showed suitable areas for the presence of *L*. *cernuum* north of 40°N (S2 Fig).

**Fig 6:**
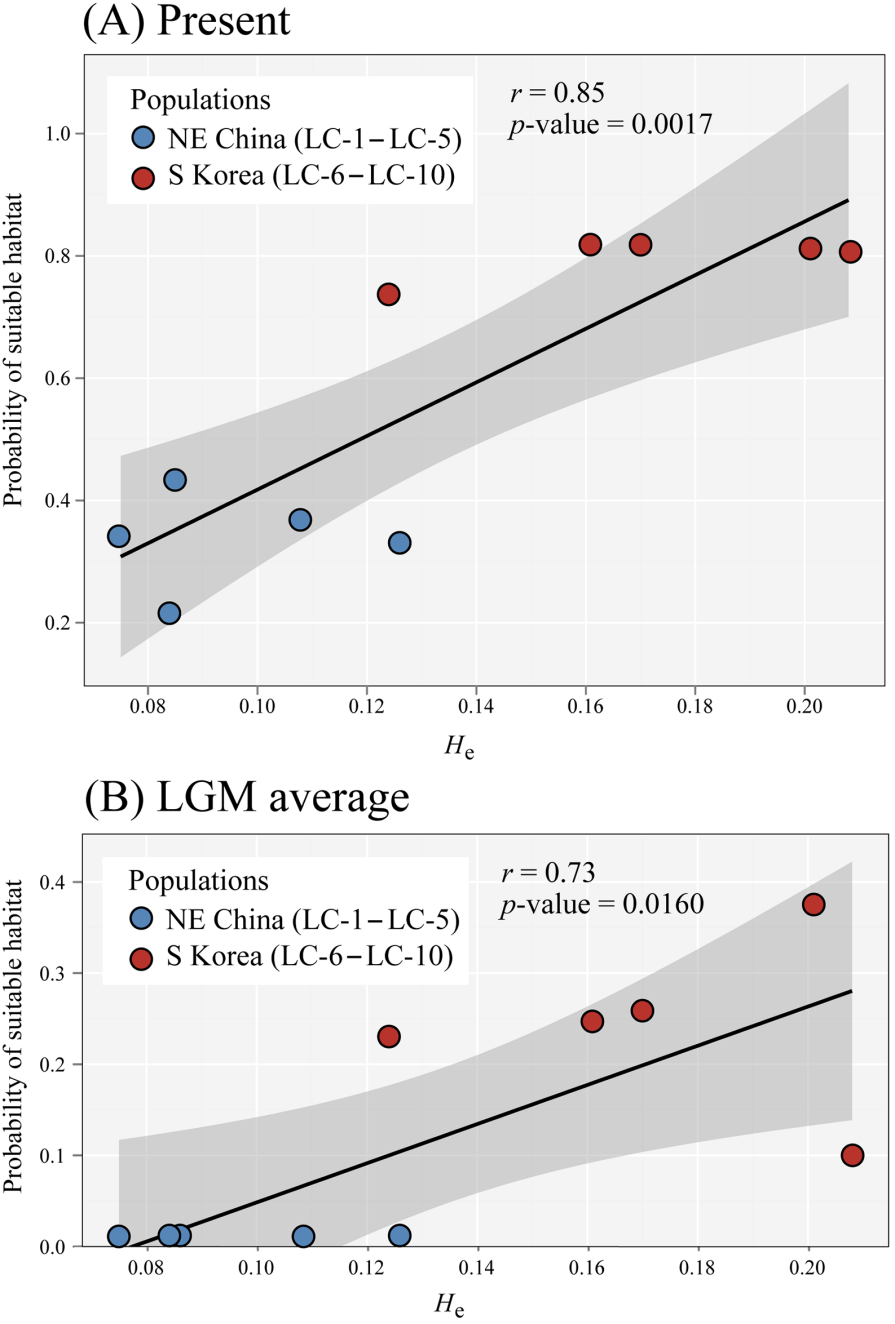
Pearson’s correlations between expected the expected heterozygosity (*H*_e_) and habitat suitability (measured as MaxEnt’s logistic output) for all *Lilium cernuum* studied individuals, for (A) the present time, and (B) the LGM.

As anticipated, ENM and genetic data showed good agreement, as there was a positive and statistically significant correlation between *H*_e_ and habitat suitability for the studied populations of *L*. *cernuum*, for both the present time and the LGM (Fig 6). Notably, there was no any northern population (NE China) that showed a higher probability of occurrence relative to southern ones (South Korea) for both time frames (Fig 6).

## Discussion

### Patterns of genetic diversity in northern and southern populations: Implications for glacial/post-glacial plant dynamics

Levels of within-population genetic diversity found in *L*. *cernuum* are in agreement with our prediction that the southern populations (those from South Korea) harbor greater genetic variation than northern ones (NE China) (%*P*_p_ = 56.7 vs. 43.3, *A*_p_ = 1.84 vs. 1.63, *AR* = 1.79 vs. 1.60, and *H*_ep_ = 0.173 vs. 0.095; Table 2). In addition, the Chinese populations contain a subset of the allelic diversity of the Korean ones; no allele is exclusive to the Chinese populations (S1 Table). Our results suggest that *L*. *cernuum* was probably not present *in situ* in NE China during the LGM and, thus, the current northern populations are the result of post-glacial recolonization from southern glacial refugia. The higher genetic polymorphism detected at the studied populations from South Korea indicates that such refugia would have been located along the BDDG, which mostly constitutes the current southern range of the species. This scenario of survival in southern refugia followed by northwards migration is consistent with the reconstructed palaeodistribution of *L*. *cernuum* for the LGM, a time when the only suitable habitats for the species would have been limited to areas below 40°N or so (Fig 5 and S2 Fig). Most of the current range of the species in South Korea (Fig 1) is included within areas of medium to high probability of occurrence in the LGM models, with the relative exception of MIROC-ESM (Fig 5). Therefore, *L*. *cernuum* would have had the chance to persist *in situ* in South Korea during the LGM and, perhaps, at other glacial maxima. In contrast, the current northern range of the species (the Chinese provinces of Jilin and Liaoning, and Primorsky Krai in Russian Far East) would not have harbored climatically suitable areas for *L*. *cernuum* during the Pleistocene glacial periods. Given the positive relationship between *H*_e_ and habitat suitability in *L*. *cernuum*, ENM might constitute a simple and cost-effective surrogate of genetic diversity studies. Although not explicitly tested, some studies with boreal/temperate plant species that are also native to our study region (Korea plus NE China and Russian Far East) also show an apparent congruence between genetic diversity levels and habitat suitability, identifying the mountains of South Korea as a putative glacial refugium [72,73]. Of these, it is especially relevant the study carried out with *Quercus mongolica* [73], the dominant species of the plant communities in which *L*. *cernuum* usually occurs.

The results from the present study are in agreement with a recently published review of the region’s Pleistocene biogeography [5], in which the authors demonstrated that the BDDG served as a Pleistocene refugium for (but not exclusively) the boreal and temperate flora of NE Asia; such statement is based on both palaeoecological and genetic evidences. In this review [5], the authors summarized the extant palaeovegetation reconstructions for NE Asia, which mostly suggest that the vegetation belts suffered major range displacements, nearly comparable to those occurred in other regions of the world even though ice sheets were not present in the vast majority of NE Asia during the LGM (see Fig 1 in [5]). In NE China and in the southern parts of the Russian Far East (i.e. Primorsky Krai), LGM vegetation was either treeless or a mixture of pure boreal forests and non-forest vegetation (tundra, shrub-tundra, or polar deserts) [20–24,74,75]. A few pollen records available for the areas that correspond to the northern range of *L*. *cernuum* also indicate that the vegetation there adapted to much colder conditions than today. For instance, in a couple of sites at latitude ca. 43°N, in the southernmost tip of Primorsky Krai, boreal forests dominated by *Betula* (accompanied by *Larix*, *Picea*, and *Pinus*) would have occurred [76]. Similarly, in the contiguous Jilin Province of China (a site located in a hilly area at ca. 100 km of the Korean border and ca. 250 km west from LC-2), vegetation probably dominated by cold deciduous and needle-leaved forests (*Betula* and *Picea* with the presence of *Abies* and *Pinus*) would have occurred [76]. Taken all together, it is highly likely that boreal/temperate herbaceous elements like *L*. *cernuum* would have not been present *in situ* in areas such as NE China or Russian Far East during the LGM.

More favorable habitats for this lily, instead, would have occurred further south. According to the tentative LGM vegetation map for the Korean Peninsula provided by [5], non-arboreal vegetation would have been limited to the northern half of North Korea, whereas the rest of the peninsula would have been covered by boreal and mixed (boreal/temperate) forests, with temperate ones at the southernmost tip. Although pollen records within or around the BDDG are very scarce, it is likely that the central to northern sections would have sustained boreal forests, whereas mixed forests were probably ubiquitous in the central to southern parts of this mountain range (see [5] and references therein for more details).

Although the results must be interpreted with caution because of the limited sampling in the northern range of *L*. *cernuum* (neither populations from Liaoning Province nor Russian Far East have been studied), there is a clear pattern of higher genetic diversity for the Korean populations compared to the Chinese ones; such results are fully compatible with the genetic studies conducted for species endemic or native to the region, which have been recently compiled by [5]. First, as noted in the Introduction, the meta-analysis of the allozyme literature published for plant species occurring on the BDDG revealed a pattern of high within-population genetic diversity (*H*_ep_ = 0.159; [5]), much larger than the “classical” values of reference (e.g. [77,78]). The value of *H*_ep_ for the Korean populations of *L*. *cernuum* (0.173) is close to the mean value reported by [5], whereas that for the Chinese populations is much lower (*H*_ep_ = 0.095). Second, the still small but growing body of studies on genetic diversity and phylogeography at regional level including populations located in southern refugia on the Korean Peninsula and populations located further north, are mostly consistent with the “southern richness vs. northern purity” pattern. That is, Korean populations harbor more genetic diversity, ancestral haplotypes, and/or significant amounts of unique haplotypes/alleles (see [5] and references therein). Very similar to our study, for example, [79] examined the genetic variation of the boreal tree *Pinus koraiensis* across the NE Asian continent (South Korea—with three of the four populations located in the BDDG, NE China and Russian Far East) by means of allozyme and RAPD markers. The authors found that levels of within-population genetic variation significantly decreased with increasing latitude. More recently, [72] found a similar pattern of latitudinal decrease of both genetic diversity and genetic singularity across NE Asia for the boreal/temperate tree *Acer mono*; both cpDNA and microsatellites suggested that the BDDG was the origin for the recolonization of NE China, a scenario also recovered from ENM [72].

### Levels of genetic diversity and structure of *L*. *cernuum* and implications for conservation

The Korean populations of *L*. *cernuum* maintain high levels of genetic variation when compared to other herbaceous perennials mainly occurring on the BDDG (Table 1 in [5]), as also reported in a previous study [27]. Therefore, Korean populations seem not to be of conservation concern, although it should be taken into account that several populations of *L*. *cernuum* in South Korea are small and discontinuously distributed along the ridge of the BDDG (M. Y. Chung and M. G. Chung, pers. obs.). However, other populations, such as LC-7 (Mt. Deokhang), would enjoy large effective population sizes (*N*_e_) [26]. The low *F*_ST_ values both at local (0.019 at a scale of <1 km; [26]), country (0.119 at a scale of 15–300 km; [27]) and regional level (0.094 at a scale of 14–730 km; present study) suggest high historical gene flow between populations. This result is an expected outcome given the role of the BDDG as Pleistocene refugium but also the species’ high potential for seed dispersal [26].

Even being relatively isolated geographically at the southern tip of the species’ distribution, the Korean population LC-10, that occurs at a high-elevation mountain within the BDDG (Mt. Sobaek, ca. 1220 m), has the lowest amount of genetic diversity among the five southern populations (but still higher than the northern populations). On the other hand, the populations LC-7 (Mt. Deokhang) and LC-8 (Yongyeon Cave), located approximately at the center of the current range for the species within South Korea, maintain the highest levels of genetic diversity among the studied populations. As mentioned above, populations around LC-7 maintain large effective population sizes on the order of hundreds [26]. These findings are in accordance with the “center-periphery” model (reviewed in [80]) which proposes that marginal populations are genetically less diverse than core ones; such model is based in the assumption that both effective population sizes (*N*_e_) and rates of gene flow (*m*) are higher at the range center compared to range margins [80]. Historical processes are increasingly recognized as major factors creating such pattern (e.g. the influence of the Pleistocene glacial/interglacial cycles on species’ demography). The low levels of polymorphism detected for the population LC-10, as also reported for the population of Mt. Gaji for the same species [27], fit well the genetic pattern expected for the “rear-edge populations” hypothesis of [81]. According to these authors, populations located at the low-latitude margins of species ranges are expected to show reduced within-population genetic variation (see also [82]) and, thus, may merit specific conservation measures.

In contrast to some populations distributed along the ridge of the BDDG, populations from NE China and Russian Far East that are close to the border with North Korea are relatively large and continuously distributed (S. Son and G. U. Suh, pers. obs.). Given present-day population sizes, population genetics theory predicts that genetic diversity should be higher in the populations from NE China compared to populations from South Korea. However, our results show the opposite pattern (most likely due to the northwards post-glacial expansion of *L*. *cernuum*), stressing the importance of empirical genetic studies in plant species [83]. In spite of their lower polymorphism compared to their southern counterparts, the studied NE Chinese populations of *L*. *cernuum* should not be regarded as genetically extremely depauperate. Reference values for plants in general (*H*_ep_ = 0.116; [78]), plants regionally-distributed (*H*_ep_ = 0.118; [77]), and short-lived herbaceous perennials (*H*_ep_ = 0.096; [77]) are only slightly higher than the observed values for the Chinese populations of *L*. *cernuum* (mean *H*_ep_ = 0.095).

If we use the equilibrium equation between *F*_IS_ and *t* (outcrossing rates) or *s* (selfing rates) (*F*_IS_ = *s*/(2 − *s*) or *F*_IS_ = (1 − *t*)/(1 + *t*); [84,85]), we obtain a *t* of 0.688 for *L*. *cernuum*, suggesting a mixed mating system in the species. The occurrence of selfing, however, might not be the only factor explaining the observed heterozygote deficiency at most populations, as other factors, including non-random mating with relatives (biparental inbreeding) and Wahlund effect (population subdivision), may account. Because NE Chinese populations of *L*. *cernuum* have been observed to be large and continuous (S. Son and G. U. Suh, pers. obs.), the higher levels of inbreeding for NE Chinese (*F*_IS_ = 0.280) compared to South Korean (*F*_IS_ = 0.136) populations are probably indicating different reproductive behavior between the two groups instead of population subdivision. Detailed field work would be necessary to see whether there is reduced pollinator service in the northern populations compared to the southern ones. The significantly larger *F*_IS_ estimates found in the NE Chinese populations compared to those in the southern ones may have conservation implications, as inbreeding may lead to reduced fitness [86].

The loss or reduction of genetic diversity is of particular concern for conservationists and managers because it may lead directly to the decrease of biological fitness of populations. Thus, maintenance of genetic diversity is often viewed as a prerequisite for adaptation to environmental changes [86] and, thus, coping with the Sixth Mass Extinction. Understanding the genetic make-up of populations occurring on the BDDG is crucial because they have been regarded as a reservoir and at the same time the genetic stock for post-glacial recolonization for temperate and boreal plant species in NE Asia [5]. Our results are consistent with such scenario, emphasizing the conservation value of southern populations, which could be used for future translocation or reinforcement actions.

## Conclusions

Our results from genetic and ENM analyses support the hypothesis that *L*. *cernuum* vanished from the Chinese provinces of Jilin and Liaoning, and Primorsky Krai in Russian Far East during the LGM, and that the species was only able to persist in southern glacial refugia, presumably located along the BDDG in South Korea. Our results are in concordance with previous studies showing that a pattern of survival in southern refugia followed by post-glacial recolonizations would have been common for the boreal and temperate flora of NE Asia [5,12]. Our results further suggest that the populations of *L*. *cernuum* in South Korea are particularly worthy of protection.

## Supporting information

S1 Fig Estimation of the most likely value of *K*, as determined by the ln Pr(*X*|*K*) and the Δ*K* statistics (left and right, respectively), with the aid of Structure Harvester. (DOCX)

S2 Fig LGM-ensemble map obtained from the projections with the three global climate models employed (CCSM4, MIROC-ESM, and MPI-ESM-P). (DOCX)

S1 Table Allele frequencies of nine polymorphic loci of the 10 populations of *Lilium cernuum*. (XLS)

S2 Table Results of statistical tests for evidence of recent population bottlenecks in *Lilium cernuum*. Numbers reported are *P* values of sign and Wilcoxon sign-rank tests conducted using the program BOTTLENECK.
(DOCX)
