# Supplementary material for: Comparison of genetic variation between northern and southern populations of Lilium cernuum (Liliaceae): Implications for Pleistocene refugia
Source: PLoS One. 2018 Jan 4;13(1):e0190520. doi: 10.1371/journal.pone.0190520 (PMC5754063; doi:10.1371/journal.pone.0190520)
Supplement: S1 Fig — (DOCX) [file pone.0190520.s001.docx]

**S1 Figure**

The most likely *K* was estimated using (**left**) the log probability of data [ln Pr( *X* | *K* )] values [53] and (**right**) the Δ*K* statistic of [54] using Structure Harvester 0.6.94 [55].





**References**

53. Pritchard JK, Wen X, Falush D. Documentation for structure software: Version 2.3. Chicago: Department of Human Genetics, University of Chicago. 2010. Available from: [http://pritch.bsd.uchicago.edu/structure_software/release_versions/v2.3.4/struct ure_doc.pdf](http://pritch.bsd.uchicago.edu/structure_software/release_versions/v2.3.4/struct%09ure_doc.pdf)

54. Evanno G, Regnaut S, Goudet J. Detecting the number of clusters of individuals using the software STRUCTURE: a simulation study. Mol Ecol. 2005; 14: 2611–2620.

55. Earl DA, vonHoldt, BM. STRUCTURE HARVESTER: a website and program for visualizing STRUCTURE output and implementing the Evanno method. Conserv Genet Resour. 2012; 4: 359–361.
