# Supplementary material for: Comparison of genetic variation between northern and southern populations of Lilium cernuum (Liliaceae): Implications for Pleistocene refugia
Source: PLoS One. 2018 Jan 4;13(1):e0190520. doi: 10.1371/journal.pone.0190520 (PMC5754063; doi:10.1371/journal.pone.0190520)

**S2 Figure**

LGM-ensemble map obtained from the projections with the three global climate models employed (CCSM4, MIROC-ESM, and MPI-ESM-P).


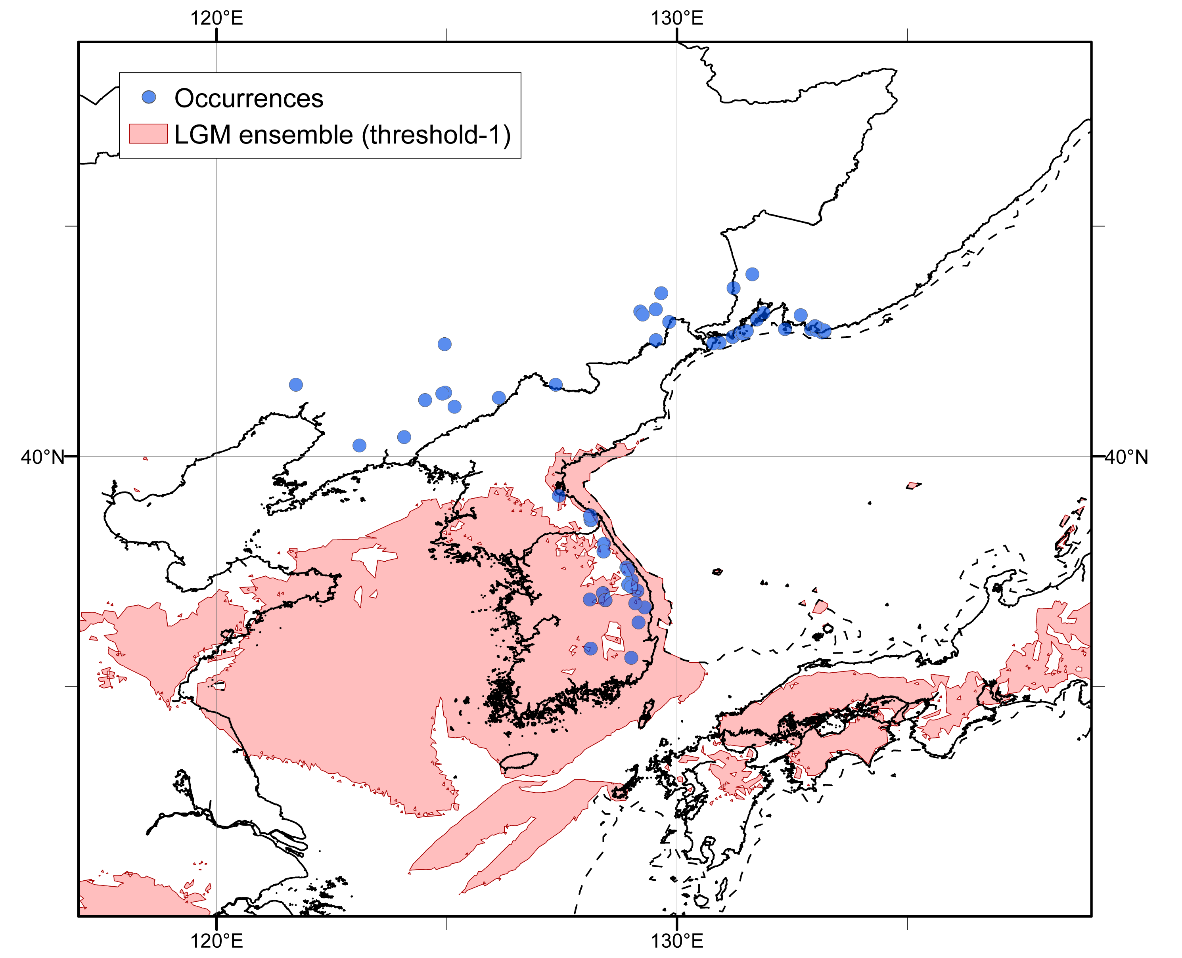

Supplement: S2 Fig — (DOCX) [file pone.0190520.s002.docx]
