# Supplementary material for: Comparison of genetic variation between northern and southern populations of Lilium cernuum (Liliaceae): Implications for Pleistocene refugia
Source: PLoS One. 2018 Jan 4;13(1):e0190520. doi: 10.1371/journal.pone.0190520 (PMC5754063; doi:10.1371/journal.pone.0190520)
Supplement: S2 Table — Numbers reported are P values of sign and Wilcoxon sign-rank tests conducted using the program BOTTLENECK. (DOCX) [file pone.0190520.s004.docx]

**S2 Table** Results of statistical tests for evidence of recent population bottlenecks in *Lilium cernuum*. Numbers reported are *P* values of sign and Wilcoxon sign-rank tests conducted using the program BOTTLENECK.

| Population | Sign test | Wilcoxon sign rank- test |
| --- | --- | --- |
| LC-1 | 0.504 | 0.688 |
| LC-2 | 0.228 | 0.922 |
| LC-3 | 0.128 | 0.922 |
| LC-4 | 0.200 | 0.984 |
| LC-5 | 0.278 | 0.891 |
| LC-6 | 0.545 | 0.766 |
| LC-7 | 0.542 | 0.406 |
| LC-8 | 0.384 | 0.410 |
| LC-9 | 0.143 | 0.078 |
| LC-10 | 0.137 | 0.945 |
